# Histologische und klinische Überschneidung von Acne inversa und Morbus Crohn unter IL-17-Inhibition

**DOI:** 10.1007/s00105-025-05545-6

**Published:** 2025-07-14

**Authors:** Lennart Ocker, Christina Scheel, Nessr Abu Rached, Martin Dörler, Markus Stücker, Falk Bechara

**Affiliations:** Internationales Zentrum für Acne inversa/Hidradenitis Suppurativa, Klinik für Dermatologie, Allergologie und Venerologie, Ruhr-Universität Bochum, Gudrunstr. 56, 44791 Bochum, Deutschland

## Anamnese

Eine 24-jährige Patientin stellte sich zur operativen Therapie einer schweren Acne inversa/Hidradenitis suppurativa (HS) vor. Seit 2 Jahren bestanden rezidivierende, schmerzhafte Hautveränderungen mit drainierenden Fistelgängen im Gluteal- und Perianalbereich sowie in geringerem Ausmaß axillär. Trotz wiederholter Abszessinzisionen, antibiotischer Therapien und einer 3‑monatigen Systemtherapie mit dem IL-17A-Antikörper Secukinumab (300 mg s.c. alle 4 Wochen) blieb eine deutliche Besserung aus. Zusätzlich berichtete die Patientin über seit ca. 4 Wochen bestehende Stuhlunregelmäßigkeiten, Hämatochezie sowie einen ungewollten Gewichtsverlust von 11 kg in den letzten 4 Monaten. Eine Koloskopie 6 Monate zuvor ergab keinen pathologischen Befund.

## Untersuchung und Diagnostik

### Klinischer Befund

**Abb. 1:**
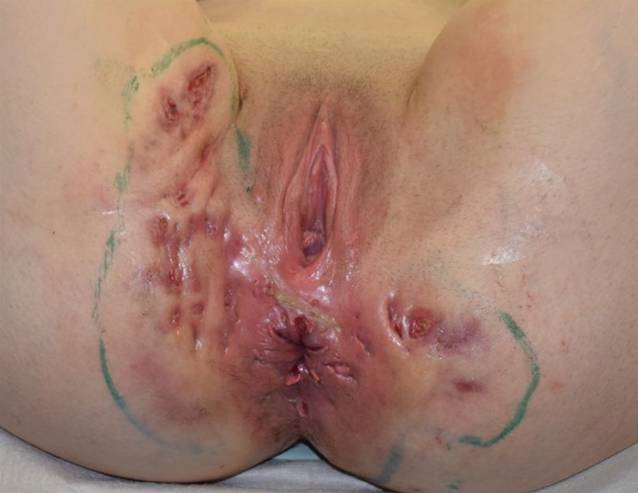
Präoperative Übersichtsaufnahme der Patientin mit markierten Resektionsgrenzen im Bereich der Genitoanalregion

In der Genitoanalregion fanden sich konfluierende, entzündliche Fistelgänge mit aktiver eitriger Drainage sowie scharf begrenzte Erosionen perianal (Abb. 1). Axillär zeigten sich gering entzündliche Knoten und solitäre, nicht drainierende Fistelgänge. Die klinische Schweregradeinschätzung der Acne inversa erbrachte ein Hurley-Stadium III, einen IHS4(International Hidradenitis Suppurativa Severity Scoring System)-Score von 47. Weiterhin berichtete die Patientin über eine ausgeprägte Einschränkung ihrer Lebensqualität, gekennzeichnet durch einen DLQI-Score von 23.

### Histopathologischer Befund

**Abb. 2:**
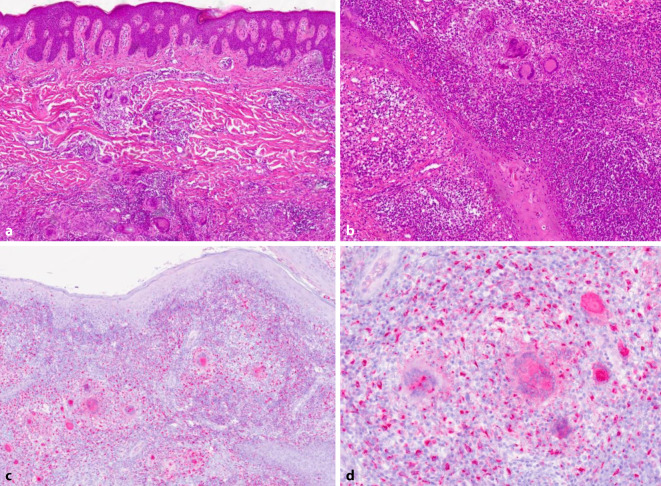
Hautpräparat der Perianalregion der Patientin. **a** Hämatoxylin-Eosin-Färbung, Vergr. 30:1. **b** Hämatoxylin-Eosin-Färbung, Vergr. 50:1. **c** Immunhistochemische Färbung mit Antikörpern gegen CD68, Vergr. 30:1. **d** Immunhistochemische Färbung mit Antikörpern gegen CD68, Vergr. 100:1

Die Untersuchung des Exzidats aus der Perianalregion zeigte unter einem akanthotisch verbreiterten Deckepithel mit Spongiose und Parakeratose fistelartige Gangstrukturen, umgeben von fibrosiertem Gewebe mit parallelisierten Kollagenfasern (Abb. 2a, b). Dermal fand sich ein dichtes, gemischtzelliges, granulomatöses Infiltrat mit zahlreichen multinukleären Riesenzellen und starker CD68-Expression ohne einen direkten follikulären Bezug (Abb. 2c, d).

## Therapie und Verlauf

Die Patientin wurde operativ mittels Exzision der irreversibel zerstörten Hautareale in der Perianalregion versorgt. Die Wundheilung erfolgte sekundär. Aufgrund der anamnestischen gastrointestinalen Beschwerden unter IL-17-Blockade und der histologischen Hinweise auf eine granulomatöse Entzündung wurde eine weiterführende gastroenterologische Abklärung veranlasst. In der Stuhldiagnostik zeigte sich eine Erhöhung des fäkalen Calprotectins mit 93 µg/g. In der endoskopischen Diagnostik bestätigte sich der Verdacht auf eine chronisch entzündliche Darmerkrankung (CED) im Sinne eines Morbus Crohn. Vor diesem Hintergrund wurde die laufende IL-17A-Inhibition mit Secukinumab beendet, und es erfolgte die Umstellung der Systemtherapie auf einen TNF-α-Inhibitor (Adalimumab, 80 mg s.c. alle 2 Wochen). Unter dieser Therapie kam es zu einem Rückgang der gastrointestinalen Beschwerden sowie zu einer Stabilisierung der kutanen Entzündungsaktivität.

## Diskussion

Die vorliegende Kasuistik unterstreicht die klinische und histopathologische Überschneidung von HS und kutaner Manifestation eines Morbus Crohn. Histopathologisch zeigt HS typische epidermale Veränderungen mit Akanthose, follikulärer Hyperkeratose und perifollikulärer gemischtzelliger Entzündung sowie epithelialisierte dermale Fistelgänge mit umgebender Fibrose im weiteren Krankheitsverlauf [1, 5]. Das Vorkommen von vereinzelten mehrkernigen Riesenzellen bei HS im Rahmen einer follikulären Fremdkörperreaktion ist gut beschrieben [10]. Der Morbus Crohn zeigt neben fistelartigen Gangbildungen typischerweise ein granulomatöses, histiozytäres Infiltrat mit reichlich multinukleären Riesenzellen, sowie häufig eine granulomatöse Lymphangitis als spezifisches Unterscheidungsmerkmal zur HS [9]. Die klinische und histopathologische Differenzierung zwischen einer HS und einer kutanen Manifestation eines Morbus Crohn bleibt somit schwierig und bedarf einer sorgfältigen interdisziplinären Abklärung.

Im vorliegenden Fall wurden bei manifester HS im Axillär- und Genitoanalbereich eine klinische Verschlechterung der Entzündungsaktivität sowie ein Neuauftreten gastroenterologischer Beschwerden unter IL-17A-Inhibition beobachtet, sodass von einer Erstmanifestation bzw. Demaskierung einer chronisch entzündlichen Darmerkrankung bei bekannter HS auszugehen ist. Assoziationen der beiden Krankheitsbilder sind mit einer Prävalenz von bis zu 17,3 % beschrieben [7].

Auch pathophysiologisch bestehen Überschneidungen in den immunologischen Mechanismen beider Erkrankungen. Eine zentrale Rolle kommt hierbei der IL-23/IL-17-Achse zu, die sowohl in der Pathogenese von HS als auch von Morbus Crohn beteiligt ist [4, 8]. Während IL-17-Inhibitoren wie Secukinumab und Bimekizumab für die Behandlung der HS zugelassen und wirksam sind, besteht bei CED das Risiko einer potenziellen Krankheitsverschlechterung unter dieser Therapie [8]. Darüber hinaus wurden auch Erstmanifestationen von CED unter IL-17-Blockade beobachtet [3, 6]. Neuere Fallserien beschreiben das Auftreten von CED mit histologischen IBD-Merkmalen unter IL-17-Inhibition teils bereits wenige Tage nach Therapiebeginn [2]. Die Wahl einer immunmodulatorischen Systemtherapie muss daher unter Berücksichtigung aller klinischen Manifestationen und potenziellen Begleiterkrankungen individuell getroffen werden.

Die Stabilisierung der gastrointestinalen Symptomatik unter Adalimumab in unserem Fall bekräftigt die Notwendigkeit einer indikationsgerechten Therapieanpassung bei gleichzeitiger dermatologischer und gastroenterologischer Erkrankung.

## Fazit für die Praxis

Die Fallbeschreibung illustriert die diagnostische Herausforderung bei HS-Patient:innen mit gastrointestinalen Symptomen und perianaler granulomatöser Infiltration. Eine kutane Manifestation einer CED, insbesondere von Morbus Crohn, ist differenzialdiagnostisch in Betracht zu ziehen. In unklaren Fällen sollten weiterführende Untersuchungen wie Entzündungsmarker im Stuhl, MRT-Bildgebung oder eine endoskopische Abklärung veranlasst werden. Der Einsatz von IL-17-Inhibitoren wird bei Verdacht auf CED nicht empfohlen. Eine interdisziplinäre Zusammenarbeit mit der Gastroenterologie ist essenziell.
